# Supplementary material for: Chromothripsis during telomere crisis is independent of NHEJ, and consistent with a replicative origin
Source: Genome Res. 2019 May;29(5):737–49. doi: 10.1101/gr.240705.118 (PMC6499312; doi:10.1101/gr.240705.118)
Supplement: Supplemental Material [file supp_gr.240705.118_Supplemental_file_1.zip › contigs/annotated_contigs/DB113/contig.2.DB113_length_625_mean_cov_6.0608.docx]

**DB113_length_625_mean_cov_6.0608**

```
CAGGAAAGCTGAACTTAGAAAACAGCTTGCCTTCTACCCTCTCACCCCACCTGCAGCATCAACATCTACAGCCTATCTTCAGGCTCCAA
 >chr10:13270141-13270563 - E=3e-239
CTCATCCAAAAGCATCAGATCACCTGAAAATTTTTATATGGTTTTCTTGTGGCTTTATGCTTCTCCTATTTGTATCCTCTTCTCTAAGC

CTTGGTCTTTTCCTTACTAGCAAAAAATAGAGAGTAGGAAAGGCCCAGGGAGGCCAGGAGTTTTGAGGGGCTGAGGTGGGCGGATCACT

TGAGGTCAGGAGTTTGAGACTAGCCTAGCGAATGTGGCGAAACCCTGTCTCTACTAAAAATAATTAAAAATTAGCCAGGCGTGATGGTG

CACACCTGTAGTCTCAGCTACTTGGGAGGCTGAGGCAGGTGAATTGCTTGAAACTTGGAGGTGGAG|AAA|TCCTGACCTCAGGTGACC
                                                                      >chr10:12362307-1
CGCCTGCCTCAGCCTCCCAAAGTGCTGGGATTACAGGCAGGAACCACCATGCCCTGCCCCTCTGCTCCTTTGCATGTGCTGTTCTTTCC
2362507 + E=1e-108
ATCTGGAATTTTCTCAGTCCTTCTCCCTGTCCATCCAGGTCCCCAGGGGTTCAAATCAAGTCCTCTCTACTCTGAGAACATCTTCCCAG

TCAA
```
